# Assessment of sexuality among women in Morocco

**DOI:** 10.1192/j.eurpsy.2022.2078

**Published:** 2022-09-01

**Authors:** I. Katir, C. Fatima Zahra, A. Korchi, S. Belbachir, A. Ouanass

**Affiliations:** 1 Ar-razi University Psychiatric Hospital of Salé, Salé, Morocco, Psychiatry, Salé, Morocco; 2 CHU Avicenne Rabat, Psychiatric Hospital Ar -razi Salé, salé, Morocco; 3 Hôpital psychiatrique ARRAZI de Salé, Psychiatrie, Salé, Morocco

**Keywords:** women, Assessment, sexuality, Morocco

## Abstract

**Introduction:**

Having a healthy active sex life is essential to maintain good physical health and offer the possibility of establishing moments of mental well-being. Until now, not many studies have addressed this health aspect in our context, while problems related to intimate relationships remain one of the most frequent causes of consultation in psychiatry.

**Objectives:**

The objectives of our study are: to assess sexuality among Moroccan women, analyze their sexual behavior with an epidemiological description, determine their sources of information, and identify the potential causes that could lead to lower their libido.

**Methods:**

We conducted a cross-sectional study with around hundreds of women in the general population using the female sexual function index (FSFI), associated with a questionnaire that includes age, place of residence, origin, marital status, number of children, profession, social status, age of first sexual experience, details of different sexual practices, sources of information related to genital life, the means of contraception and the presence of comorbidities.

**Results:**

Preliminary results show a limited understanding of sexuality among women of low socioeconomic status. Women with a high level of education are more fulfilled and this is due to the ease of access to information and care. Depression contributes greatly to lower libido and marital conflict.

**Conclusions:**

Sexuality remains today one of the most taboo subjects in our country and more among the female population. Hence the need for sex education begins with self-knowledge, understanding of different practices, and psychological support for all women from a young age toward enduring a healthy flourishing sex life.

**Disclosure:**

No significant relationships.

